# C/EBPβ-LIP mediated activation of the malate-aspartate shuttle sensitizes cells to glycolysis inhibition

**DOI:** 10.1016/j.molmet.2023.101726

**Published:** 2023-04-14

**Authors:** Tobias Ackermann, Hidde R. Zuidhof, Christine Müller, Gertrud Kortman, Martijn G.S. Rutten, Mathilde J.C. Broekhuis, Mohamad Amr Zaini, Götz Hartleben, Cornelis F. Calkhoven

**Affiliations:** 1 European Research Institute for the Biology of Ageing (ERIBA), University Medical Center Groningen, University of Groningen, 9700 AD Groningen, the Netherlands; 2 iPSC CRISPR Facility, University Medical Center Groningen, University of Groningen, 9700 AD Groningen, the Netherlands

**Keywords:** C/EBPβ, Malate-aspartate shuttle, Glycolysis, NAD^+^, Cancer

## Abstract

**Objective:**

Cancer cells use glycolysis for generation of metabolic intermediates and ATP needed for cell growth and proliferation. The transcription factor C/EBPβ-LIP stimulates glycolysis and mitochondrial respiration in cancer cells. We initially observed that high expression of C/EBPβ-LIP makes cells vulnerable to treatment with the glycolysis inhibitor 2-deoxyglucose. The aim of the study was to uncover the involved mechanisms of C/EBPβ-LIP induced sensitivity to glycolysis inhibition.

**Methods:**

We used genetically engineered cell lines to examine the effect of C/EBPβ-LIP and -LAP protein isoforms on glycolysis and NADH/NAD^+^ metabolism in mouse embryonic fibroblasts (MEFs), and triple negative breast cancer (TNBC) cells that endogenously express high levels of C/EBPβ-LIP. Analyses included assays of cell proliferation, cell survival and metabolic flux (OCR and ECAR by Seahorse XF96). Small molecule inhibitors were used to identify underlying metabolic pathways that mediate sensitivity to glycolysis inhibition induced by C/EBPβ-LIP.

**Results:**

The transcription factor C/EBPβ-LIP stimulates both glycolysis and the malate-aspartate shuttle (MAS) and increases the sensitivity to glycolysis inhibition (2-deoxyglucose) in fibroblasts and breast cancer cells. Inhibition of glycolysis with ongoing C/EBPβ-LIP-induced MAS activity results in NADH depletion and apoptosis that can be rescued by inhibiting either the MAS or other NAD^+^-regenerating processes.

**Conclusion:**

This study indicates that a low NADH/NAD+ ratio is an essential mediator of 2-deoxyglucose toxicity in cells with high cytoplasmic NAD^+^-regeneration capacity and that simultaneous inhibition of glycolysis and lowering of the NADH/NAD^+^ ratio may be considered to treat cancer.

## Highlights

• Cellular expression of C/EBPβ-LIP induces dependence on glycolysis and sensitizes cells to 2-deoxyglucose.
• C/EBPβ-LIP stimulates NAD^+^-regeneration through the malate aspartate shuttle.
• Inhibition of NAD^+^-regenerating processes reduces cell toxicity of 2-deoxyglucose in C/EBPβ-LIP expressing cells.

## 1 Introduction

Cancer cells reprogram their metabolism to support *de novo* synthesis of macromolecules that are needed for cell growth and proliferation [1]. Most prominently, cancer cells increase glucose uptake and metabolize glucose by aerobic glycolysis, which was first recognized by Otto Warburg (reviewed in [2]). Later, it was shown that cancer cells maintain high rates of both glycolysis and oxidative phosphorylation (OXPHOS) to meet the high demand of energy and substrates for anabolic processes [3,4]. The high glycolytic flux provides the cancer cell with macromolecules by uncoupling glycolysis from the mitochondrial tricarboxylic acid (TCA) cycle and diverting glucose carbon into biosynthetic pathways, including the pentose phosphate pathway, hexosamine pathway and serine biosynthetic pathway [5]. During glycolysis and serine biosynthesis NAD^+^ serves as an electron acceptor for reactions catalyzed by glyceraldehyde-3-phospate dehydrogenase (GAPDH) and phosphoglycerate dehydrogenase (PHGDH), and is thereby reduced to NADH. Consequently, the NADH/NAD+ ratio in cancer cells is usually very high [6]. To sustain a high glycolytic flux and allow serine biosynthesis, NAD^+^ must be regenerated. The mitochondria are major sites of NAD^+^-regeneration by complex I of the electron transport chain (ETC), where NADH-derived electrons contribute to oxidative respiration and ATP production [7]. Yet, NAD^+^ and NADH cannot pass the mitochondrial membranes and therefore cells use substrate cycles that transport the electrons derived from oxidation of cytosolic NADH into the mitochondria. One such cycle is the malate-aspartate shuttle (MAS), which uses malate as intermediate electron carrier to transport the electrons from cytosolic NADH to mitochondrial NADH [8]. The other cycle is the glycerol-phosphate shuttle (GPS), which uses Glycerol-3-phosphate as intermediate to transport the electrons over the membrane to mitochondrial FADH that enters the ETC at complex II (Supplemental Fig. 1A). In either way the carbon flux of the glycolysis is coupled to mitochondrial function [9,10]. There are early reports that cancer cells depend on the MAS for biomolecule synthesis and proliferation, while this is less clear for the GPS [11,12]. When mitochondrial NAD^+^ regeneration does not suffice, which is mostly the case for the high glycolytic flux in cancer cells, cytosolic NAD^+^ is regenerated in addition through reduction of pyruvate into lactate by the enzyme lactate dehydrogenase (LDH) (the Warburg effect) [13]. Yet, LDH activity alone cannot provide sufficient cytosolic NAD^+^, and through lactate secretion glucose carbon is lost for biomolecule synthesis [10].

The transcription factor CCAAT/enhancer binding protein beta (C/EBPβ) is known to regulate organismal metabolism [14,15]. The CEBPB-mRNA is translated into three protein isoforms; two transcriptional activators C/EBPβ-LAP1 and -LAP2 (also named LAP∗ and LAP) and the N-terminally truncated protein isoform and transcriptional inhibitor C/EBPβ-LIP (hereinafter referred to as LAP and LIP) [16,17]. LIP expression is tightly controlled by the mTORC1-4E-BP pathway and involves a *cis*-regulatory upstream open reading frame (uORF) in the CEBPB-mRNA leader sequence [16,18]. Overexpression of LIP induces cellular transformation [16] and increases tumor incidence in mice [19], while LIP deficiency reduces tumour incidence in mice [20]. Moreover, LIP is highly expressed in breast cancer, ovarian cancer, colorectal cancer, and anaplastic large cell lymphoma [[21], [22], [23], [24], [25], [26]]. Recently, we demonstrated that LIP induces cancer metabolism with increased glycolysis and mitochondrial respiration through regulation of the let-7/LIN28B circuit [27].

Here, we show that stimulation of the MAS in high-LIP expressing cells results in dependence on glycolysis for NADH/NAD^+^ homeostasis and cell viability. Inhibition of glycolysis in cells with high-LIP expression results in depletion of NADH and low NADH/NAD^+^ ratios as the MAS continues to oxidize cytosolic NADH into NAD^+^. This condition is associated with apoptosis, which can be rescued by inhibition of the MAS or other NAD^+^-regenerating processes. Our data indicate that low NADH/NAD^+^ ratios are toxic for cancer cells and that metabolic reprogramming by LIP or through otherwise increased MAS activity makes cancer cells vulnerable to glycolytic inhibitors.

## 2 Methods

### 2.1 Cell culture

The Cebpb-knockout MEFs were isolated from Cebpb-knockout mice (Cebpbtm1Vpo/J, Jackson Laboratory stock no: 006873, mixed background: 129S& MF1) and immortalized by p19ARF-knockdown using a p19ARF-shRNA pSuper-Retro vector and puromycin (1.5 μg/ml) selection. BT20 cells, MDA-MB-231 and all immortalized MEF cell lines were culture in high glucose DMEM supplemented with 10% FBS, 10 mM HEPES, 1 mM Sodium Pyruvate and 100U/ml Penicillin Streptomycin. BT549, ZR-75-1, T47D and MCF7 breast cancer cells were maintained in RPMI1640 medium supplemented with 10% FBS, 25 mM HEPES, 1 mM Sodium Pyruvate and 100U/ml Penicillin/Streptomycin. *Cebpb*-ko MEF were described before [14].

### 2.2 DNA constructs

Plasmids containing rat C/EBPβ-LAP, rat C/EBPβ-LIP and human C/EBPβ-LIP and were described before (Zidek et al., 2015). For CRISPR/Cas9 mediated knockout of both C/EBPβ isoforms, guide RNA sequences (LAP: 5′-GAGTGGCCAACTTCTACTACG-3′, LIP: 5′-GCGCTTACCTCGGCTACCAGG-3′) were cloned into pSpCas9(BB)-2A-puro (PX459) v2.0 (http://www.addgene.org/62988/).

### 2.3 Transfection

Immortalized MEFs were transfected with an empty, rat C/EBPβ-LIP or -LAP containing pcDNA3 or pSV2Zeo vector by using FugeneHD (Promega) according to the manufactures protocol. For stable overexpression, C/EBPβ-ko MEFs were treated with 0.2 mg/ml Zeocin (Invitrogen). To maintain the expression cells were culture with 0.1 mg/ml Zeocin in the medium. T47D cells and MCF7 cells were transfected with empty or human LIP-containing pcDNA3.1 via Fugene HD (Promega) using the manufactures protocol. For stable expression, MCF7 cells were selected with 0.8 mg/ml, T47D with 0.4 mg/ml G418. For CRISPR/Cas9 mediated knock out of both C/EBPβ isoforms, BT20 cells were transfected with Fugene HD (Promega) according to the manufactures protocol and selected with puromycine (1 μg/ml). After the selection, clones were grown out and C/EBPβ level were analyzed by western blot.

### 2.4 Proliferation assays

To determine the proliferation and survival of cancer cells, relative cell numbers were measured using the CellTiter-Fluor™ Cell Viability Assay (Promega) after 3 days of treatment. Measurements were performed according to the supplier’s manual.

### 2.5 Metabolic flux analysis

Metabolic flux analysis was performed using a Seahorse XF96 Extracellular Flux analyzer (Agilent Bioscience). 1.5 × 10^4^ EV, LAP or LIP overexpressing *Cebpb*-ko MEFs were seeded 4h before the assay. Assays were performed according to the manufactures protocol. Injected drugs were oligomycin (2.5 μM) for blockage of ATP related respiration, dinitrophenol (50 μM) to uncouple the mitochondrial membrane (maximum respiration) and 2-DG (100 mM) for inhibition of glycolysis. 3 × 10^4^ EV and LIP overexpressing T47D cells were seeded 16 h before the experiments. Assays were performed according to the manufactures protocol. Injected drugs were UK5099 (5 μM) for blockage of mitochondrial pyruvate transporter, BPTES (3 μM) for blockage of glutaminase, etomoxir (40 μM) for blockage of fatty acid transport into the mitochondrion, 2-DG (100 mM) for inhibition of glycolysis, Rotenone (4 μM) blockage of complex 1 and oligomycin (2.5 μM) for blockage of ATP related respiration. To measure the activity of the MAS, cells were permeabilized by injection of saponin (25 μg/ml) and the substrates of the MAS (Glutamate (1 mM) and Malate (2 mM)) were added separately by single injections.

### 2.6 Luciferase based assays

NADH, NAD^+^, ATP and ADP level were distinguished using luciferase assays. 24 h before the assay, 7500 cells per well were seeded in a 96-well plate. Experiments were performed according to manufactures protocols (NADH/NAD+: Promega, G9071; ATP/ADP: Biovision, K255-200). For detection, a GloMax-Multi Detection System (Promega) was used.

Caspase3/7 activity was measured 3 days after the treatment with a commercially available kit (Caspase-Glo 3/7 Assay, Promega).

### 2.7 Immunoblot analysis

Cells and tissues were lysed using RIPA buffer. Equal amounts of protein were separated via SDS-PAGE and transferred to a PVDF membrane using Trans-Blot Turbo System (Bio-Rad). The following antibodies were used for detection: C/EBPβ (E299) from Abcam, α-Tubulin (GT114) from GeneTex and β-actin (clone C4) (#691001) from MP Biomedicals. For detection, HRP-conjugated secondary antibodies (Amersham Life Technologies) were used. The signals were visualized by chemiluminescence (ECL, Amersham Life Technologies) using ImageQuant LAS 4000 mini biomolecular imager (GE Healthcare Bioscience AB) and the supplied software was used for the quantification of the bands.

### 2.8 Statistical analysis

IC50 and r2 values were calculated using a non/linear fitting algorithm (log[inhibitor] vs response - variable slope (four parameters)) in Graphpad Prism.

## 3 Results

### 3.1 LIP renders cells dependent on glucose metabolism

**Figure 1:**
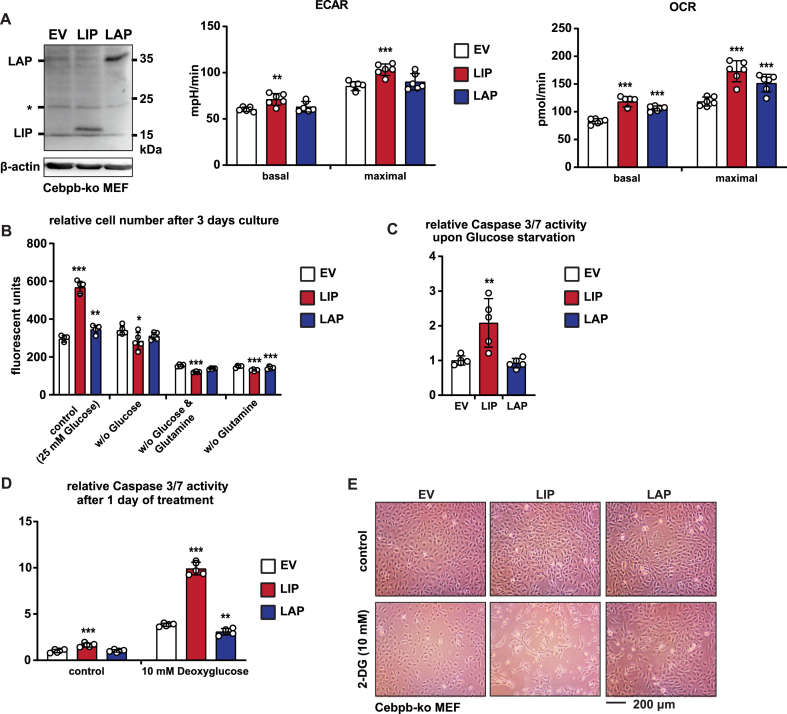
LIP induces reliance on glycolysis for cell proliferation and survival. (A) The immunoblot at the left shows expression of LAP, LIP and β-actin as loading control in *Cebpb*-ko fibroblasts transfected with expression vectors for LAP, LIP or empty vector (EV) control. The bar graphs show values of ECAR and OCR associated with LAP, LIP or EV expression (n = 6). (B) Relative cell numbers of *Cebpb*-ko fibroblasts transfected with expression vectors for LAP, LIP or empty vector (EV) control after 3 days of culture in media with or without glucose and/or glutamine (n = 5). (C) Relative Caspase3/7 activity in *Cebpb*-ko fibroblasts transfected with expression vectors for LAP, LIP or empty vector (EV) control after 3 days of culture in medium without glucose (n = 5). (D) Relative Caspase3/7 activity of *Cebpb*-ko fibroblasts transfected with expression vectors for LAP, LIP or empty vector (EV) control after one day of treatment with 2-DG (n = 5). (E) Microscopic pictures of *Cebpb*-ko fibroblasts transfected with expression vectors for LAP, LIP or empty vector (EV) control after one day of treatment with 2-DG. Statistical differences were analyzed by Student’s t-tests. Error bars represent SD, ∗p < 0.05, ∗∗p < 0.01, ∗∗∗p < 0.001.

We examined the C/EBPβ isoform specific effects on cellular metabolism by measuring the extracellular acidification rate (ECAR) as an indicator for glycolytic flux, and the oxygen consumption rate (OCR) as a measure for mitochondrial metabolism (Seahorse XF96). Experimental re-expression of LIP in immortalized *Cebpb*-knockout MEFs increased both basal and maximal ECAR, while expression of LAP had no effect on the ECAR (Figure 1A and Supplemental Figure 1B-D). The LIP-induced increase in ECAR was abrogated by treatment with 2-deoxyglucose (2-DG), which inhibits glycolysis (Supplemental Figure 1E), Together these data show that LIP enhances the glycolytic flux. In addition, expression of LIP and to a lesser extent LAP increased the OCR in the cells (Figure 1A and Supplemental Figure 1E) indicating that both LIP and LAP stimulate mitochondrial metabolism with different potential. Next, we investigated whether LIP-expressing cells depend on the increased glycolytic flux for cell proliferation and survival. Withdrawal of glucose resulted in a strong decrease in viable LIP-expressing cells after three days of culture compared to cells cultured in the presence of glucose (25 mM) (Figure 1B). Glucose deprivation did not result in altered cell numbers for LAP-expressing cells or empty vector (EV) control cells compared to glucose containing media (Figure 1B). Deprivation of the alternative carbon source glutamine alone or together with glucose resulted in a strong decrease in cell numbers in all three cell lines (EV, LIP, LAP), confirming a general requirement of glutamine for cell proliferation independent of LIP or LAP expression (Figure 1B). Furthermore, upon glucose starvation Caspase 3/7 activity is more strongly induced in LIP- compared to LAP-expressing or EV cells suggesting higher levels of apoptosis in LIP-expressing cells (Figure 1C). In line with the glucose-deprivation experiments, treatment with the glycolytic inhibitor 2-DG abrogates LIP-induced increase in ECAR in a similar manner like for EV and LAP expressing cells (Supplemental Figure 1C). 2-DG treatment drives LIP-expressing cells into apoptosis as was shown by a strong increase in Caspase 3/7 activity (Figure 1D) and microscopy (Figure 1E), while LAP-expressing and control (EV) cells survive under this condition. Hence, these data show that proliferation and survival of LIP-expressing MEFs highly depends on glucose metabolism.

**Figure 2:**
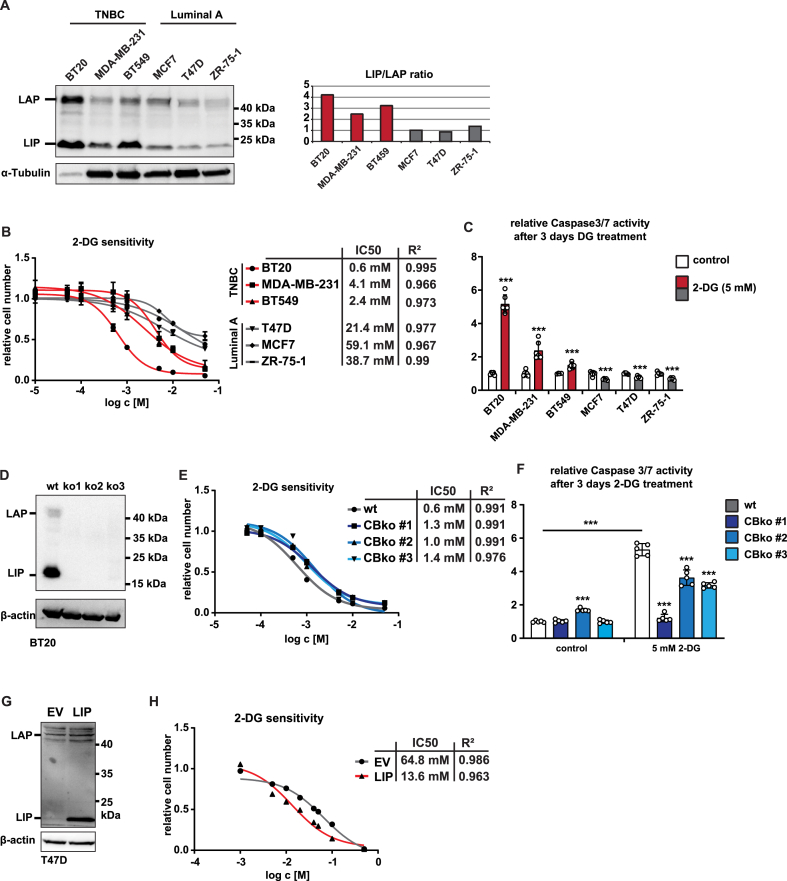
High LIP/LAP ratios render TNBC cell lines sensitive to 2-DG. (A) Immunoblot showing expression of LAP, LIP and α-tubulin as loading control in the TNBC BT20, MDA-MB-231, BT549, and Luminal A MCF7, T47D, ZR-75–1 breast cancer cell lines with quantified LIP/LAP ratios at the right. (B) Dose–response-curve of the six breast cancer cell lines mentioned in A after 3 days of 2-DG treatment (n = 5). (C) Relative Caspase 3/7 activity of the six breast cancer cell lines mentioned in A after 3 days of 2-DG treatment (n = 5). (D) Immunoblot showing expression of LAP, LIP and β-actin as loading control in wt BT20 cells and three clones of CRIPR/Cas9 derived *CEBPB*-ko BT20 cells. (E) Dose–response-curve of wt BT20 and *CEBPB*-ko BT20 cells after 3 days of 2-DG treatment (n = 5). F) Relative Caspase 3/7 activity in wt BT20 and *CEBPB*-ko BT20 cells after 3 days of 2-DG treatment (n = 5). (G) Immunoblot showing expression of LAP, LIP and β-actin as loading control in T47D cells transfected with expression vectors for LIP or empty vector (EV) control. (H) Dose–response-curve of T47D cells expressing LIP or EV control and after 3 days of 2-DG treatment (n = 5). Statistical differences were analyzed by Student’s t-tests. Error bars represent SD, ∗p < 0.05, ∗∗p < 0.01, ∗∗∗p < 0.001. IC50 values of dose–response are determined by R-squared (R^2^) statistical analysis.

As a cellular model we chose breast cancer cell lines since high expression levels of LIP have been described for aggressive breast cancer types [21]. Immunoblot analysis of a panel of breast cancer cell lines revealed high endogenous LIP expression (high LIP/LAP ratio) in triple negative breast cancer (TNBC) subtype, while low LIP expression with lower LIP/LAP ratios were found in luminal A subtype breast cancer cell lines (Figure 2A). To examine whether high LIP expression is associated with higher sensitivity to inhibition of glycolysis we generated dose-response curves for 2-DG treatment and cell multiplication. From the TNBC cell lines, the BT20 cells that express the highest LIP levels were most sensitivity to 2-DG (IC50=0.6 mM), followed by BT549 (IC50=2.4 mM) and MDA-MB-231 (IC50=4.1 mM) (Figure 2B and Supplemental Figure 2A). The luminal A type cells with low LIP expression showed a poorer response to 2-DG treatment (IC50>20 mM) (Figure 2B and Supplemental Figure 2A). Furthermore, the sensitivity to 2-DG correlated with increased Caspase3/7 activity as a measurement for apoptosis in BT20 cells and to lesser extent in BT549 and MDA-MB-231 cells, while Luminal A breast cancer cells even showed a slight decrease in Caspase3/7 activity in response to 2-DG (Figure 2C). To address whether the high sensitivity for 2DG of BT20 cells depends on C/EBPβ expression we generated *CEBPB*-ko BT20 cells by CRISPR/Cas9 genome editing (Figure S2B). In three independent knockout clones (Figure 2D) the sensitivity to 2-DG was reduced as measured by cell multiplication (Figure 2E and Supplemental Figure 2C) and by a significant reduction in Caspase 3/7 activation, although to various extents (Figure 2F). Next, we investigated whether overexpression of LIP in the low LIP-expressing T47D and MCF-7 Luminal A cell lines would result in increased sensitivity to 2-DG, as measured by cell multiplication. The higher LIP expression indeed increased the sensitivity to 2-DG by 5-fold for T47D and 2.3-fold for MCF7 (Figure 2G, H and Supplemental Figure 2D, E). However, LIP overexpression did not induce Caspase 3/7 activity in T47D cells suggesting that, either in TNBC cells other factors contribute to 2-DG-induced apoptosis in addition to LIP, or that in the Luminal A cells mechanisms are active that prevent apoptosis (Supplemental Figure 2F). Taken together, these data show that high levels of LIP expression render TNBC cells dependent on glycolysis for cell proliferation and survival.

### 3.2 LIP increases the use of glycolysis derived NADH for mitochondrial respiration

**Figure 3:**
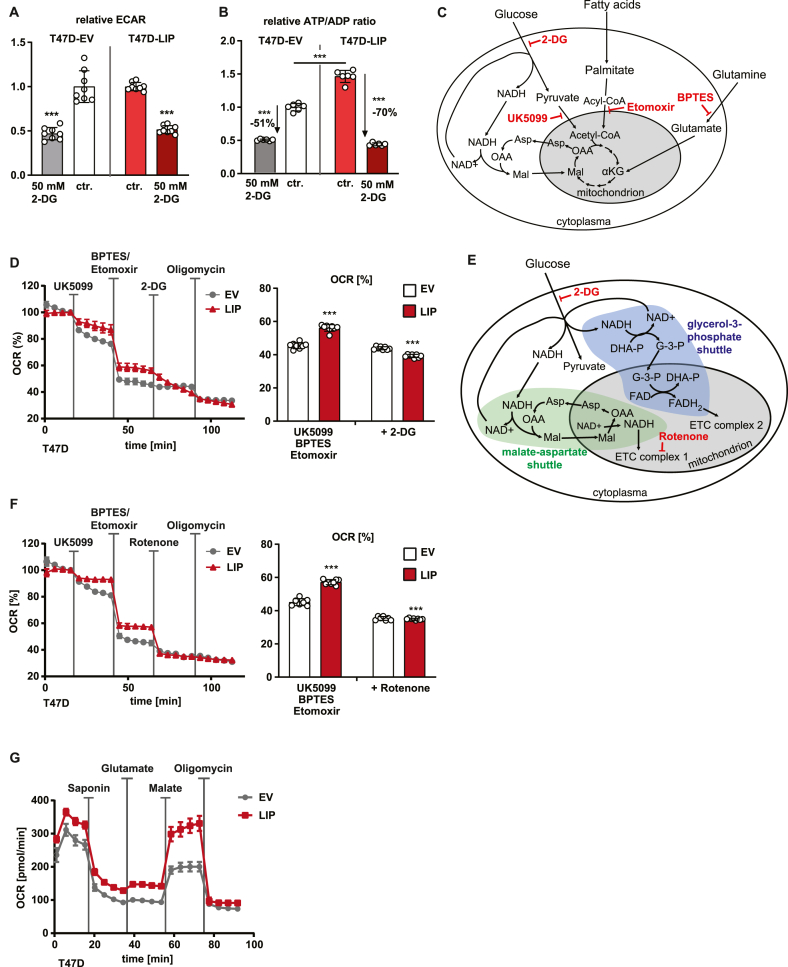
LIP stimulates the MAS and its usage of cytoplasmic NADH. (A) Relative ECAR of LIP expressing or control (EV) T47D cells before and 10 min after with treatment with 2-DG (n = 5). (B) Relative ATP/ADP ratios in LIP expressing or control (EV) T47D cells after one day of 2-DG treatment or without treatment (ctr.) (n = 5). (C) Schematic representation of the flow of metabolites between cytoplasm and the mitochondria and inhibitors of specific pathways. (D) OCR of LIP expressing or control (EV) T47D cells with subsequent injection of UK5099, BPTES plus Etomoxir, 2-DG and oligomycin (n = 6). Bar graph at the right shows a different representation of the data (n = 6). (E) Schematic representation of the MAS and the GPS, the flow of involved metabolites and used inhibitors. (F) OCR of LIP expressing or control (EV) T47D cells with subsequent injection of UK5099, BPTES plus Etomoxir, rotenone and oligomycin (n = 6). Bar graph at the right shows a different representation of the data (n = 6). (G) OCR of LIP expressing or control (EV) T47D cells with subsequent injection of saponin (permeabilization of cell membrane), glutamate, malate and oligomycin (n = 6). Statistical differences were analyzed by Student’s t-tests. Error bars represent SD, ∗∗∗p < 0.001.

We next asked why high LIP expression increases the dependence on glycolysis. We first examined whether ATP/ADP ratios change upon LIP-induction, which could be involved in inducing apoptosis [28]. One source of ATP production in cancer cells is the high glycolytic flux. Inhibition of glycolysis with 2-DG reduced the ECAR as a measure for glycolytic flux to the same extent in T47D-LIP cells and control T47D cells (Figure 3A). The expectation was that ATP/ADP ratios would decline to comparable degrees. However, compared to the 51% reduction in T47D-EV cells 2-DG treatment more strongly reduced the ATP/ADP ratio in the T47D-LIP cells with 70% (Figure 3B). Thus, in T47D-LIP cells, in addition to the ATP produced during aerobic glycolysis other glucose-dependent pathway(s) contribute for 19% to the production of cellular ATP, which if prevented would compromise cell proliferation and/or viability. We reasoned that a mechanism involving glycolysis-coupled stimulation of mitochondrial respiration and the associated generation of ATP in the electron transport chain (ETC) might explain the extra ATP production induced by LIP.

Mitochondrial metabolism is fueled mainly by pyruvate (as a source for Acetyl-CoA) and NADH from glycolysis, Acyl-CoA from fatty acid catabolism, and α-Ketoglutarate from glutaminolysis (Figure 3C). The possible engagement of the individual pathways can be studied by measuring changes in oxygen consumption rate (OCR; Seahorse XF96) upon treatment with specific pathway inhibitors (Figure 3D). Inhibition of the mitochondrial pyruvate carrier with the drug UK5099 reduced the OCR in T47D-LIP cells to a lesser extent compared to control T47D cells, suggesting that pyruvate from glycolysis is not a critical metabolite to fuel respiration in T47D-LIP cells (Figure 3C). When in addition the use of palmitate and glutamine by the mitochondria is blocked with Etomoxir and BPTES (Bis-2-5-phenylacetamido-1,3,4-thiadiazol-2-ylethyl sulfide) the T47D-LIP cells still maintained higher OCR compared to the T47D-EV cells (Figure 3D). Under these conditions the NADH generated by glycolysis can still fuel mitochondrial respiration. Therefore, we used 2-DG to inhibit glycolysis on top of the treatment with UK5099/Etomoxir/BPTES and found the OCR of T47D-LIP cells now decreased to a similar extent than control T47D cells (Figure 3D,E). These data show that LIP stimulates the usage of cytosolic glycolysis derived NADH for promoting mitochondrial respiration.

### 3.3 LIP stimulates the malate-aspartate shuttle

The two pathways that transport electrons derived from cytosolic NADH into the mitochondria are the malate-aspartate shuttle (MAS) and the glycerol-phosphate shuttle (GPS), rotenone abrogated the difference in OCR caused by LIP expression in T47D cells, indicating that MAS is the critical pathway involved in enhancing mitochondrial respiration (Figure 3F). Next, we examined the capacity of the MAS in T47D-LIP versus control T47D cells by experimentally applying exogenous malate after cell membrane permeabilization using saponin. Permeabilization of the cell membrane resulted in a strong reduction of the OCR for both cell lines as substrates for mitochondrial respiration are leaking out of the cell (Figure 3G). The subsequent supply of glutamate slightly increased the OCR in both cell lines while the supply of the MAS substrate malate resulted in a strong increase in OCR in the T47D-LIP cells and to a much lesser extent in the control T47D cells (Figure 3G), which was reduced to the same level by treatment with rotenone (Figure 3F). These data show that LIP stimulates the MAS, transporting electrons from cytoplasmic NADH into the mitochondria, to stimulate mitochondrial respiration.

### 3.4 Altered NADH usage causes apoptosis in LIP overexpressing cells

**Figure 4:**
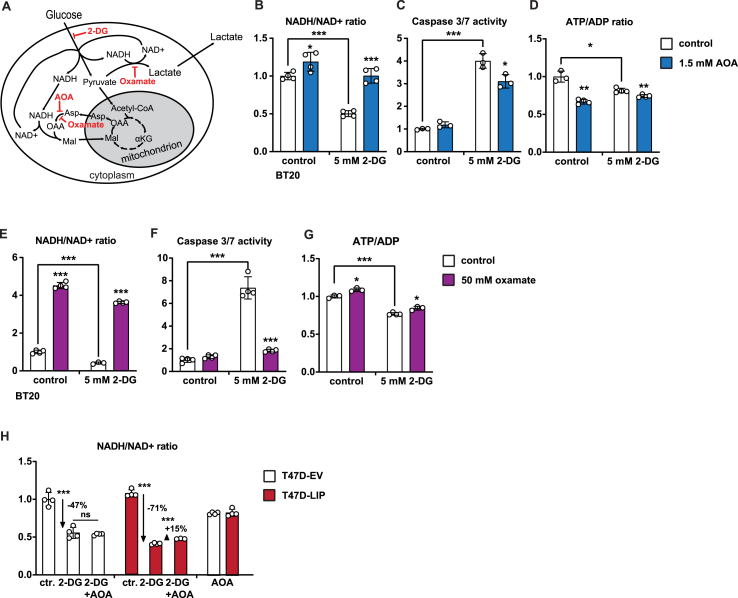
Inhibition of NADH-consuming processes reduces toxicity of 2-DG. (A) Schematic representation of cytoplasmic NADH-consuming processes MAS and LDH and their inhibitors AOA and oxamate, respectively, and the glycolytic inhibitor 2-DG. (B) Relative NADH/NAD^+^ ratios in BT20 cells after 1 day of treatment with solvent (control), 2-DG, AOA, or 2-DG plus AOA (n = 4). (C) Relative caspase3/7 activity of BT20 cells after 3 days of treatment with solvent, 2-DG, AOA or 2-DG plus AOA (n = 4). (D) Relative ATP/ADP ratios in BT20 cells after 1 day of treatment with solvent, 2-DG, AOA, or 2-DG plus AOA (n = 4). (E) Relative NADH/NAD^+^ ratios in BT20 cells after 1 day of treatment with solvent, 2-DG, oxamate or 2-DG plus oxamate (n = 4). (F) Relative caspase3/7 activity of BT20 cells after 3 days of treatment with solvent, 2-DG, oxamate or 2-DG plus oxamate (n = 4). (G) Relative ATP/ADP ratios in BT20 cells after 1 day of treatment with solvent, 2-DG, oxamate or 2-DG plus oxamate (n = 4). (H) Relative NADH/NAD+ ratios in T47D-LIP overexpressing or T47D-EV (empty vector) control cells after treatment of solvent (ctr.), 2-DG or AOA (n = 4). Statistical differences were analyzed by Student’s t-tests. Error bars represent SD, ∗p < 0.05, ∗∗p < 0.01, ∗∗∗p < 0.001.

Next, we asked whether the LIP-induced increase in MAS renders the cells sensitive to inhibition of glycolysis. Hypothetically, inhibition of glycolysis may lead to very low NADH/NAD^+^ ratios with potentially detrimental effects on cell viability, particularly in cells with high LIP expression because the LIP-driven MAS continues to convert cytosolic NADH into NAD^+^. Inhibition of glycolysis in the high-LIP BT20 cells with 2-DG resulted in a strong decrease in NADH/NAD^+^ ratio (Figure 4B) and induction of apoptosis (Caspase 3/7 activity) (Figure 4C). Aiming to rescue cell viability, cells were treated with aminooxyacetic acid (AOA) that broadly inhibits transaminases including the aspartate aminotransferase of the MAS (Figure 4A). Treatment with AOA fully restored the cellular NADH/NAD ratio in 2-DG treated cells (Figure 4B) and resulted in a significant although not full decrease in apoptosis (Figure 4C). The incomplete rescue from apoptosis may be due to AOA being a “promiscuous” drug, inhibiting all transaminases in the cell, which potentially may affect cell survival in addition [29]. Notably, the ATP/ADP ratio in the 2-DG treated cells did not increase upon AOA treatment (Figure 4D) and therefore cannot contribute to the increase in cell viability. In the TNBC cell lines BT549 and MDA-MB-231 that have lower LIP expression 2-DG treatment likewise lowered the NADH/NAD^+^, although to a lesser extent then in BT20 cells (Supplemental Figure 3A and D). Also, in these cell lines subsequent inhibition of the MAS with AOA restored the cellular NADH/NAD^+^ ratio, resulted in a significant decrease in apoptosis (Supplemental Figure 3B and E), and did not alter the ATP/ADP ratio in the 2-DG treated cells (Supplemental Figure 3C and F). Therefore, the data suggest that LIP-stimulated MAS activity depletes NADH from the cytoplasm and when NADH is not continuously replenished by a high glycolytic flux the low NADH/NAD^+^ ratios compromise cell viability. Oxamate is a structural analogue of pyruvate that inhibits LDH and and in a side reaction also inhibits the MAS enzyme aspartate aminotransferase (AAT) to a lesser extent (Figure 4A) [29,30]. Treatment of BT20 cells with oxamate greatly increased NADH/NAD^+^ ratios in both untreated and 2-DG treated cells, reflecting the inhibition of the NADH-consuming reactions of LDH and AAT (Figure 4E). Importantly, the strong upregulation of apoptosis measured by caspase 3/7 activity following 2-DG treatment was almost completely reverted by the NADH/NAD^+^ ratio restoring oxamate treatment (Figure 4F). In this case the ATP/ADP ratio slightly increased in the 2-DG and oxamate treated cells although not reaching control levels (Figure 4G).

Altogether, these data show that cells do not tolerate inhibition of glycolysis together with active NAD^+^-regenerating processes, and that cells can be rescued from apoptosis by restoring NADH/NAD^+^ ratios through inhibition of NAD^+^-regenerating pathways. In further support that high-LIP/high-MAS contributes to the toxicity of 2-DG is that treatment with 2-DG resulted in a stronger drop in NADH/NAD^+^ ratios (−71%) in T47D-LIP cells compared to control T47D cells (−47%) (Figure 4H). Subsequent inhibition of the MAS by AOA restored the NADH/NAD^+^ ratio with +15% in T47D-LIP but not in T47D-EV cells, showing that LIP overexpression induces the MAS in these luminal A breast cancer cells similar to the high endogenous LIP expression in TNBC cell lines.

## 4 Discussion

The data presented in this study suggest that C/EBPβ-LIP not only induces glycolysis but in addition secures continuation of the glycolytic flux by stimulating the regeneration of cytoplasmic NAD^+^ through the MAS. The coordination of these two interdependent processes that are particularly important for cancer cell growth and proliferation has, to our knowledge, not been shown for other oncogenic factors. Upon glucose withdrawal or inhibition of glycolysis with 2-DG the ongoing LIP-induced MAS strongly decreases the cellular NADH/NAD^+^ ratio, inducing apoptosis. In cells with high endogenous levels of LIP the sensitivity to 2-DG can be diminished by depletion of C/EBPβ, inhibition of the MAS or other NAD^+^-regenerating processes. Hence, LIP may mark cancer cells for sensitivity to therapeutic strategies targeting glycolysis (e.g. 2-DG).

Although, the collective data points to LIP as the causing factor of 2-DG sensitivity coupled to stimulation of the MAS a potential role of LAP could not be ruled out. Experimental shifting to a high LAP/LIP ratio by inducible LAP overexpression or CRISPR/Cas9 removal of the *cis*-regulatory upstream open reading frame (uORF) required for LIP expression [16] caused strongly reduced cell numbers and apoptosis ruling out further experiments. This suggests that high LAP expression reduces cell proliferation and survival by different means. In the context of a possible therapeutic target, we must take into account that LIP seems to act as an oncogene with pleiotropic functions. In two additional studies we have shown that LIP expression is required for TNBC cell migration [31], and that LIP stimulates glycolysis through regulation of LIN28B/let-7 pathway [27].

The MAS is a key process in the cell to connect metabolic pathways in the mitochondria and the cytoplasm and has been linked to cancer metabolism [8], for example in pancreatic cancer or non-small lung cancer where amplification of Malate dehydrogenase 1 (MDH1) was detected [32]. MDH1 is the key enzyme in the MAS that catalyzes NADH/NAD^+^-dependent reversible oxidation of malate into oxaloacetate in the cytosol. In lung cancer cells, the enhanced MAS activity is required as an alternative to LDH-catalyzed NAD^+^ generation, since glucose carbons are shuttled into biosynthetic pathways and therefore the pyruvate supply to LDH is not sufficient to regenerate NAD^+^ to maintain a high glycolytic flux [12]. The MAS allows cancer cells to efficiently use the glycolysis for both energy production and anabolic processes and thereby can stimulate proliferation or survival upon glutamine starvation [33]. Our data indicate that LIP induces an increase in MAS activity that may be part of the oncogenic activities of LIP [19,27]. To interfere with the activity of the MAS we treated cells with the general transaminases inhibitor aminooxyacetic acid (AOA). To more specifically study the involvement of individual MAS enzymes and transporters future experiments aim to examine the effects of knocking out or down the various MAS genes. Our preliminary experiments on regulation of the key factors SLC25A11 (malate/α-ketoglutarate carrier), SLC25A12 and SLC25A13 (citrin, glutamate/aspartate carrier), MDH1 (malate dehydrogenase, cytoplasmic), MDH2 (mitochondrial), GOT1 (glutamic oxaloacetic aminotransferase, cytoplasmic) and GOT2 (mitochondrial) as well as on the methylation status of MDH1 known to regulate its function did not show LIP-dependent changes. Therefore, further studies will concentrate on discovery of the underlying molecular mechanisms of LIP-MAS regulation.

We do not know how a decreased NADH/NAD^+^ ratio induces apoptosis in the cells with high LIP expression. NAD^+^ is an essential co-factor for metabolic reactions and a substrate for reactions in cell signaling pathways, including sirtuins and poly-adenosine ribose-polymerase (PARP) [34,35]. Increased NAD^+^ levels rather protect from apoptosis through sirtuin mediated mechanisms [36]. Additionally, it has been shown that NADH can bind to the apoptosis inducing factor (AIF), inducing its dimerization, and thereby maintaining the AIF-dimers in the mitochondria preventing apoptosis [37]. Low levels of NADH results in AIF monomerization and the AIF monomers can leave the mitochondria and translocate into the nucleus to induce apoptosis [38]. However, the induced MAS activity would remove NADH from the cytosol but not from the mitochondria suggesting a different, yet to be identified mechanism. Although we could not clarify the mechanism of apoptosis induction by 2-DG treatment in cells with high LIP expression, we demonstrate in this manuscript that the activation of the MAS may become an “Achilles heel” for cancer cells upon glucose restriction. We show that upon glucose starvation or inhibition of glycolysis a simultaneously active MAS creates low NADH/NAD+ ratios that are toxic to the cells.

New attempts in cancer therapy try to exploit cancer cell specific metabolic dependencies to specifically kill cancer cells. So far, cancer therapy with 2-DG is only tested for a few specific cancer types or in combination with other chemotherapeutic drugs [39]. Our study suggests that high LIP expression may be used as a biomarker for effectiveness of 2-DG or other glycolysis inhibiting drugs in cancer treatment. Further work is needed to evaluate the predictive power of LIP expression for 2-DG treatment success. Furthermore, we identified the NADH/NAD^+^ ratio as an important mediator of 2-DG toxicity *in vitro*. More experiments are required to evaluate whether 2-DG lowers the NADH/NAD^+^ ratio in tumors or whether artificial oxidation of NADH with small compounds will make cancer cells sensitive to 2-DG treatment [7].

Taken together, we found that LIP renders cells sensitive to 2-DG treatment by stimulating the MAS and its use of cytoplasmic NADH. We describe that the consequently low NADH/NAD^+^ ratio is an important mediator of 2-DG induced cell death in triple negative breast (TNBC) cancer cells. Furthermore, we suggest a new model for 2-DG sensitivity in which a low NADH/NAD^+^ ratio mediated by high LIP/high MAS or other hypothetical mechanisms drive cells into apoptosis.

## Author contributions

T.A. designed and performed the research, and collected and analyzed the data; C.M, H.R.Z., G.K, M.B. and M.A.Z. performed research and collected data; G.H. and C.F.C. designed research and supervised the project; T.A. and C.F.C. wrote the manuscript.

## Data Availability

Data will be made available on request.
