# Supplementary material for: C/EBPβ-LIP mediated activation of the malate-aspartate shuttle sensitizes cells to glycolysis inhibition
Source: Mol Metab. 2023 Apr 14;72:101726. doi: 10.1016/j.molmet.2023.101726 (PMC10160650; doi:10.1016/j.molmet.2023.101726)
Supplement: Multimedia component 1 — Supplemental Figures.pdf [file mmc1.pdf]

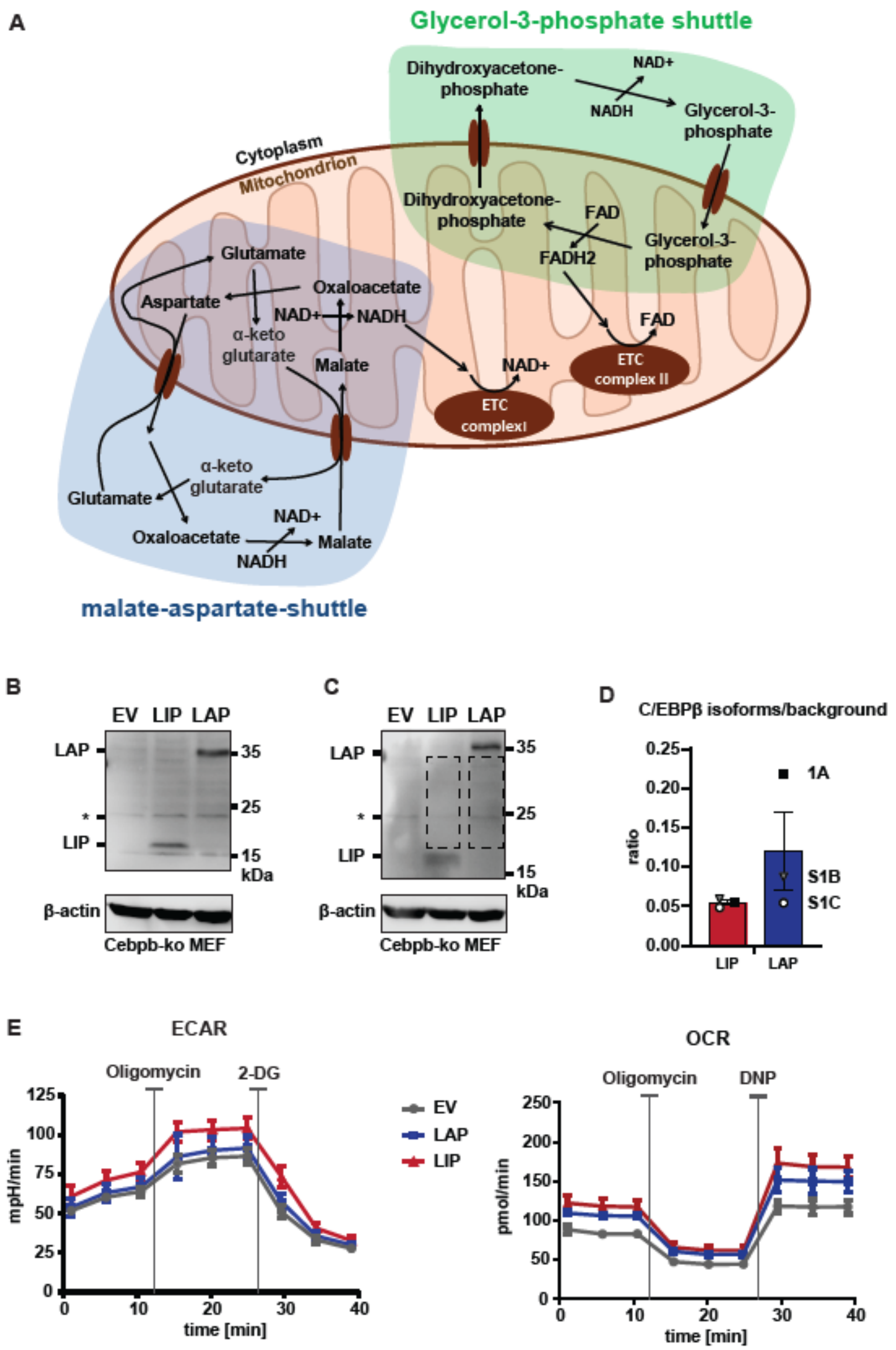


**Supplemental Figure 1**. (**A**) Schematic representation of malate-aspartate-shuttle (MAS) and glycerol-3-phosphate-shuttle (GPS). (**B**) and (**C**) immunoblots supplementary to Figure 1A showing expression of LAP, LIP and β-actin as loading control in *Cebpb*-ko fibroblasts transfected with expression vectors for LAP, LIP or empty vector (EV) control. (**D**) The bar graphs show expression levels LAP or LIP of immunoblots shown in panels B and C, and in Figure 1A compared to background. The used background areas are shown for panel C. (**E**) ECAR OCR of *Cebpb*-ko fibroblasts transfected with expression vectors for LAP, LIP or empty vector (EV) control (n=6).

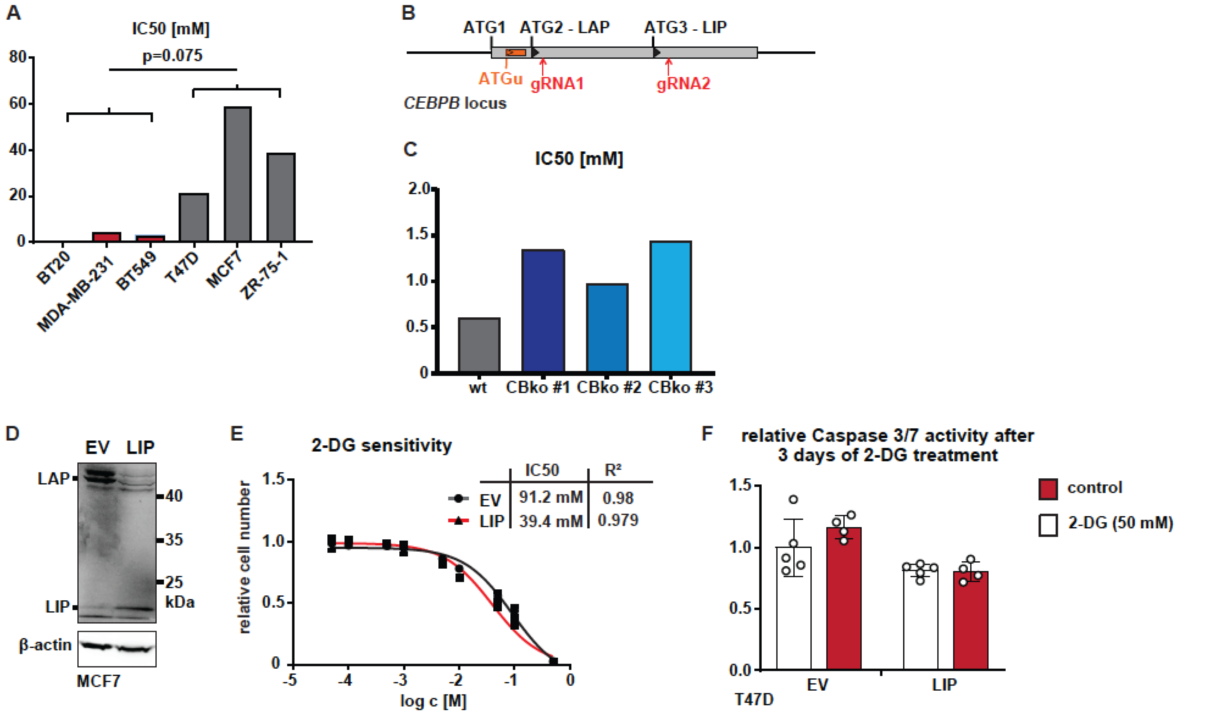


**Supplemental Figure 2**. (**A**) Bar graph representation of IC50 values of 2-DG treatment of the TNBC BT20, MDA-MB-231, BT549, and Luminal A MCF7, T47D, ZR-75-1 breast cancer cell lines (related to Figure 2B). (**B**) Schematic representation of *CEBPB* locus and the CRISPR/Cas9 based knockout strategy. The arrows mark the guide-RNAs used. (**C**) Bar graph representation of IC50 values of 2-DG treatment of wt BT20 cells and three clones of CRIPR/Cas9 derived *CEBPB*-ko BT20 cells (related to Figure 2E). (**D**) Immunoblot showing expression of LAP, LIP and β-actin as loading control in MCF7 cells transfected with expression vectors for LIP or empty vector (EV) control. (**E**) Dose-response-curve MCF7 cells expressing LIP or empty vector (EV) control after 3 days of 2-DG treatment (n=5). (**F**) Relative Caspase 3/7 activity of EV control and LIP overexpressing T47D cells after 3 days of 2-DG treatment (n=5). IC50 values of dose-response are shown with statistical analysis in $R^2$.

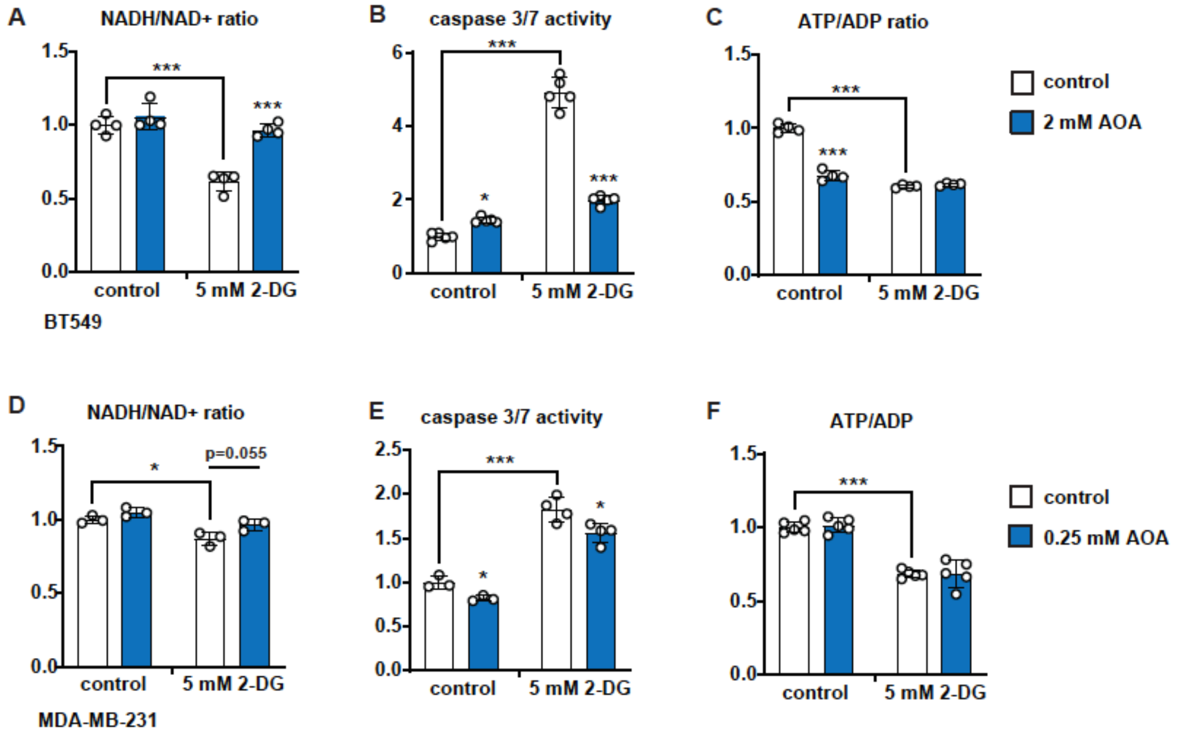


**Supplemental Figure 3.** (**A**) Relative NADH/NAD+ ratios in BT549 cells after 1 day of treatment with solvent (control), 2-DG, AOA, or 2-DG and AOA (n=4). (**B**) Relative caspase3/7 activity of BT549 cells after 3 days of treatment with solvent, 2-DG, AOA, or 2-DG and AOA (n=4). (**C**) Relative ATP/ADP ratios in BT549 cells after 1 day of treatment with solvent, 2-DG, AOA, or 2-DG and AOA (n=4). (**D**) Relative NADH/NAD+ ratios in MDA-MB-231 cells after 1 day of treatment with solvent, 2-DG, AOA, or 2-DG and AOA (n=3). (**E**) Relative ATP/ADP ratios in MDA-MB-231 cells after 1 day of treatment with solvent, 2-DG, AOA, or 2-DG and AOA (n=3). (**F**) Relative caspase3/7 activity in MDA-MB-231 cells after 3 days of treatment with solvent, 2-DG, AOA, or 2-DG and AOA (n=4). (**G**) Relative NADH/NAD+ ratios T47D cells expressing LIP or empty vector (EV) control after 1 day of treatment with solvent, 2-DG, AOA or 2-DG and AOA (n=4). Statistical differences were analyzed by Student's t-tests. Error bars represent SD, * p<0.05, *** p<0. 001.
